# Peroneal Nerve Palsy Caused by Proximal Fibular Solitary Osteochondroma: Case Report and Literature Review

**DOI:** 10.1155/2022/5865040

**Published:** 2022-09-13

**Authors:** Takashi Kozu, Masayoshi Machida, Katsuaki Taira, Noboru Oikawa, Naho Nemoto, Kazuyoshi Nakanishi

**Affiliations:** ^1^Department of Orthopaedic Surgery, Saitama Children Medical Center, Japan; ^2^Department of Orthopaedic Surgery, Nihon University, Japan

## Abstract

Osteochondroma is a relatively common benign tumor of the bone, and compressive neuropathies due to osteochondroma are comparatively rare. Here, we present a rare case of osteochondroma of the fibular head that caused peroneal nerve palsy in an 8-year-old girl. Physical examination revealed 0/5 tibialis anterior, 1/5 extensor hallucis longus, and 1/5 peroneal brevis muscle power—according to the manual muscle testing grading system, as well as numbness on the lateral side of the right leg and the back of the foot. Radiological examination and ultrasound revealed a bone tumor in the head of the right fibula. Magnetic resonance imaging ruled out spinal nerve root compression. It was discovered that the bone tumor in the fibular head had compressed and displaced the common peroneal nerve. The patient underwent surgical decompression of the right peroneal nerve. A bone region measuring 22 × 14 × 8 mm was removed. Three months postoperatively, the preoperative neurological deficits were found to be nearly resolved. The patient presented with a foot drop for 1 year, but symptoms resolved 3 months after surgery. Conventional wisdom states that surgery should be performed within 3 months, but we recommend that surgery be performed as soon as diagnosis is made even in cases with a long history, as it may improve patient symptoms and outcomes.

## 1. Introduction

Osteochondroma is a relatively common benign tumor of the bone, accounting for 10–15% of all bone tumors [1]. It is often asymptomatic, and compressive neuropathies due to osteochondroma are very rare, presenting in <1% of cases. John et al. reported 15 patients with peroneal mononeuropathy in 1158 patients studied by electromyography; of these, only one patient showed peroneal neuropathy due to multiple bony exostoses [2]. The present report describes an 8-year-old child with common peroneal nerve (CPN) palsy caused by a solitary osteochondroma of the fibular head.

## 2. Case Presentation

An 8-year-old girl visited our hospital with complaints of difficulty in walking and gradual weakness of right foot dorsiflexion for 1 year. She reported no history of trauma or neurological disease.

**Figure 1:**
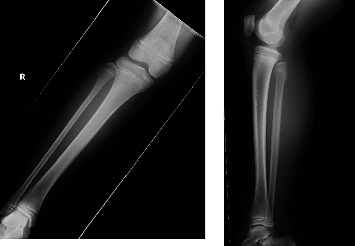
Radiograph of the right knee showing osteochondroma of the fibular head.

**Figure 2:**
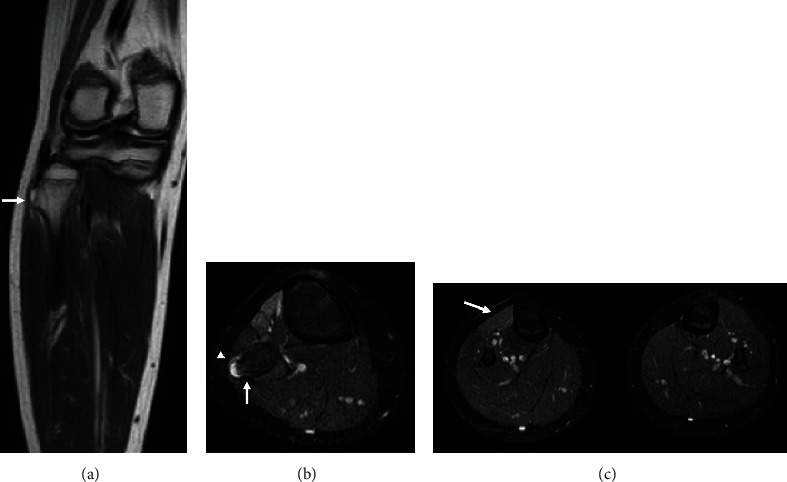
T2-weighted magnetic resonance imaging (MRI) showing (a, b) osteochondroma of the fibular head compressed peroneal nerve (white arrow: osteochondroma; white triangle: peroneal nerve) and (c) atrophy of the tibias anterior (white arrow: tibias anterior).

Neurological examination revealed 0/5 tibialis anterior, 1/5 extensor hallucis longus, and 1/5 peroneal brevis muscle power—according to the manual muscle testing grading system. And the patient had moderate sensory deficits in the anterolateral and dorsum of the foot and numbness of the lateral of the right leg and back of the foot. A Tinel-like sign was observed from the posterior part of the fibula head to the distal lower leg. X-ray and ultrasound detected an osteophyte protuberance in the fibular head (Figure 1). Magnetic resonance imaging (MRI) showed compression of the CPN caused by the bone region located at the fibular head and atrophy of the tibias anterior (Figure 2). We also ruled out spinal nerve root compression using MRI.

**Figure 3:**
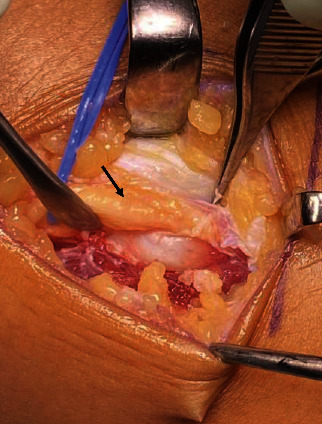
Peroneal nerve appearing compressed with adhesion caused by osteochondroma at the fibular head and arching peroneus longus muscle fibres (arrow: peroneal nerve).

The patient underwent surgical decompression of the right CPN (Figure 3), which revealed an osteophyte of the fibular head that had compressed and displaced the CPN. A bone region measuring 22 × 14 × 8 mm was removed. Postoperative pathological examination confirmed the diagnosis of osteochondroma. The patient was discharged with a foot orthotic device to prevent falling down and undertaken rehabilitation of active and passive motion in the ankle for improving range of motion and function immediately postoperative.

Three months postoperatively, the preoperative neurological deficit was nearly resolved.

## 3. Discussion

The predisposition to CPN entrapment is thought to be due to anatomical causes [3]. The nerve is superficial and covered only by subcutaneous tissue and fat at the fibular head. It wraps around the neck of the fibula and runs under the arching peroneus longus muscle fibres. This osteofibrous tunnel forms a site of potential nerve entrapment [3]. Gocmen et al. reported that CPN palsy due to osteochondroma was caused by extrinsic pressure and circumferential ring formation [4]. In the present case, the CPN was compressed by the osteochondroma at the fibular head and arching peroneus longus muscle fibres, as mentioned in previous reports. The CPN also severely adhered to the surrounding soft tissue. We believe that the manifestation of clinical symptoms was due to the location of the tumor rather than its size. The tumor size in this study was smaller than that reported previously [5]. Therefore, we hypothesized that the presence of the osteochondroma just proximal to the arching peroneus longus muscle fibres increased nerve tension and caused neuropathy.

Cherrad et al. reported a case of peroneal nerve palsy caused by proximal fibular osteochondroma (PFO) and direct trauma of the left knee's lateral side that happened during a soccer match [3]. There was exostosis in this case, but it was not triggered by trauma.

Cardelia et al. reported six patients diagnosed with PFO associated with peroneal nerve palsy [5]. The patients were treated with surgical excision between 0.5 and 24 months after onset, with four patients recovering completely and two partially. However, Birch et al. recommended early surgical intervention (<3 months), including peroneal nerve exploration with isolation of the nerve and its branches around the PFO followed by careful excision of the PFO, when neurological symptoms occur [6]. Two cases treated with surgical excision after over one year from the onset were reported by Cardelia et al. [5]. One case aged 10 years was recovered after 18 months from onset. In contrast, one case aged 13 years was not recovered completely after 24 months from onset. Although we may need to emphasize speculative character, it may be important how old the onset of the disease occurred. Therefore, we hypothesized that younger age of onset would be more likely to recover. The patient aged eight years in the present case presented with foot drop for one year, and the symptoms were completely resolved three months after surgery. In previous reports, thorough decompression and confirmation of the nerve is thought to have improved the peroneal nerve palsy after one year of diagnosis. Birch et al. reviewed previous literature and suggested that higher age (over 12 years) seemed to be associated with poorer postoperative symptoms [6]. Moreover, Wang et al. reported that, compared with adults, children with spinal cord injuries are considered to have a greater potential for neurological improvement [7]. These findings support our hypothesis.

Therefore, while surgery should ideally be performed within three months of symptom onset, it should also be performed soon after diagnosis in cases with a long history for symptomatic improvement, and it is important to undergo rehabilitation immediately postoperatively. Shah et al. reported that postoperative physiotherapy can protect against muscle atrophy, prevent secondary muscle tightness, and preserve ankle mobility [8]. We therefore hypothesize that early range-of-motion training and muscle strength training are important for nerve recovery.

We diagnosed the patient based on the findings of clinical and imaging examinations. X-rays revealed the presence of osteochondroma in the fibular head. MRI can be useful for confirming edematous changes and inflammatory findings around the peroneal nerve. Ultrasounds may also prove useful for dynamic evaluation.

## Data Availability

Data sharing is not applicable to this article as no datasets were generated or analysed during the current study.
